# A decade of EGFR inhibition in EGFR-mutated non small cell lung cancer (NSCLC): Old successes and future perspectives

**DOI:** 10.18632/oncotarget.4254

**Published:** 2015-06-12

**Authors:** Alessandro Russo, Tindara Franchina, Giuseppina Rosaria Rita Ricciardi, Antonio Picone, Giuseppa Ferraro, Mariangela Zanghì, Giuseppe Toscano, Antonio Giordano, Vincenzo Adamo

**Affiliations:** ^1^ Medical Oncology Unit AOOR Papardo-Piemonte & Department of Human Pathology, University of Messina, Messina, Italy; ^2^ Sbarro Institute for Cancer Research and Molecular Medicine, Center for Biotechnology, Temple University, Philadelphia, Pennsylvania, USA

**Keywords:** EGFR mutations, third generation EGFR TKIs, non small cell lung cancer, tyrosine kinase inhibitors, targeted therapy

## Abstract

The discovery of Epidermal Growth Factor Receptor (EGFR) mutations in Non Small Cell Lung Cancer (NSCLC) launched the era of personalized medicine in advanced NSCLC, leading to a dramatic shift in the therapeutic landscape of this disease. After ten years from the individuation of activating mutations in the tyrosine kinase domain of the EGFR in NSCLC patients responding to the EGFR tyrosine kinase inhibitor (TKI) Gefitinib, several progresses have been done and first line treatment with EGFR TKIs is a firmly established option in advanced EGFR-mutated NSCLC patients. During the last decade, different EGFR TKIs have been developed and three inhibitors have been approved so far in these selected patients. However, despite great breakthroughs have been made, treatment of these molecularly selected patients poses novel therapeutic challenges, such as emerging of acquired resistance, brain metastases development or the need to translate these treatments in earlier clinical settings, such as adjuvant therapy.

The aim of this paper is to provide a comprehensive review of the major progresses reported so far in the EGFR inhibition in this molecularly-selected subgroup of NSCLC patients, from the early successes with first generation EGFR TKIs, Erlotinib and Gefitinib, to the novel irreversible and mutant-selective inhibitors and ultimately the emerging challenges that we, in the next future, are called to deal with.

## INTRODUCTION

**Figure 1:**
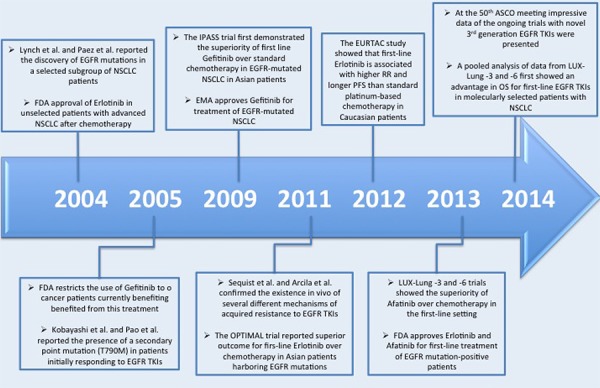
Timeline of the major progresses in the last decade in EGFR-mutated NSCLCs

The discovery of Epidermal Growth Factor Receptor (EGFR) mutations in NSCLC launched the era of personalized medicine in advanced Non Small Cell Lung Cancer (NSCLC), leading to a dramatic shift in the therapeutic landscape of this disease from a “*one size fits all*” approach to the treatment selection on molecular characteristics of tumors [1, 2]. After ten years from the individuation of activating mutations in the tyrosine kinase domain of the EGFR in NSCLC patients responding to the EGFR tyrosine kinase inhibitor (TKI) Gefitinib, several progresses have been done and first line treatment with EGFR TKIs is a firmly established option in advanced EGFR-mutated NSCLC patients. During the last decade, different EGFR TKIs have been developed and three inhibitors (Gefitinib, Erlotinib and, more recently, Afatinib) have been approved so far in these selected patients (Figure 1).

However, despite great breakthroughs have been made, treatment of these molecularly selected patients poses novel therapeutic challenges, such as emerging of acquired resistance, brain metastases development or the need to translate these treatments in earlier clinical settings, such as adjuvant therapy.

The aim of this paper is to provide a comprehensive review of the major progresses reported so far in the EGFR inhibition in this molecularly-selected subgroup of NSCLC patients, from the early successes with first generation EGFR TKIs, Erlotinib and Gefitinib, to the novel irreversible and mutant-selective inhibitors and ultimately the emerging challenges that we, in the next future, are called to deal with.

### The discovery of EGFR mutations

The Epidermal Growth Factor Receptor (EGFR) is a tyrosine kinase (TK) receptor that is activated upon binding to the Epidermal Growth Factor and other growth factor ligands, triggering several downstream pathways, including RAS/MAPK, PI3K/Akt and STAT that regulate different cellular processes, including DNA synthesis and proliferation. EGFR signaling is commonly deregulated in cancer through different mechanisms, including genetic mutations of the receptor [2, 3]. Mutant forms of EGFR have different trafficking compared with the *wild type* receptor, since some of the regulatory proteins that balance the EGFR pathway present altered expression in cancer [4].

In 2004 two different groups simultaneously identified the presence of somatic mutations in the tyrosine kinase domain of the EGFR in a small group of patients with NSCLC responding to the EGFR tyrosine kinase inhibitor (TKI) Gefitinib [5, 6]. These somatic mutations were associated with *in vitro* sensitivity to Gefitinib and with clinic-pathological characteristics preliminary associated with clinical activity [7, 8]: Asian ethnicity, female sex, adenocarcinoma histology and never smoking status. In addition, EGFR mutations were also associated with TTF-1 expression [9]. These somatic mutations mainly target the exons 18–21 of the gene, which encodes part of the TK domain of the EGFR (encoded by exons 18–24) and are clustered around the ATP-binding pocket of the receptor. The most common and best characterized EGFR mutations are in-frame deletions in exon 19, which eliminates the conserved motif LREA (residues 747–750), and the exon 21 L858R substitutions, that together constitute ~80–90% of all EGFR mutations in NSCLC. These mutations are commonly referred as “*activating mutations*” as they are constitutive activated and oncogenic. These mutant kinases present a reduced affinity for the ATP, accounting for the increased sensitivity to EGFR TKIs compared with the *wild type* counterparts, since these inhibitors compete with ATP for binding to the catalytic site [10–12].

With the exception of PI3KCA mutations [13], the majority of oncogenic drivers in NSCLC are usually mutually exclusive, including EGFR mutations.

Some authors have suggested a differential sensitivity to EGFR TKIs for exon 19 deletions and exon 21 L858R point mutations, with the former associated with longer overall survival (OS) and progression-free survival (PFS) [14, 15]. These preliminary observations were confirmed in clinical trials [16–18], although others have did not find any correlation [19, 20]. Recent meta-analyses addressed this question and reported that patients harboring exon 19 deletions are associated with a reduced progression risk than those with exon 21 point mutations [21–23] and a longer OS [22, 23]. However, the exact mechanism of this association remains largely elusive and might involve differential sensitivity to EGFR TKIs, different mechanism of acquired resistance as well as different frequency of compound mutations [21]. These data have important clinical consequences since stratification for the type of EGFR mutation might represent an important factor to consider in clinical trials with EGFR TKIs.

Oncogene addicted tumors, such as EGFR mutated NSCLCs, may present peculiar patterns of metastatization compared with *wild type* tumors, including a more frequent liver involvement at the diagnosis [24], higher tendency to central nervous system metastatization [25–27] and higher likelihood of brain metastases detection at first presentation [28] diffuse and/or miliary pulmonary metastases [28, 29]. However, others did not find any differences in brain and bone metastases development between EGFR-mutated patients and *wild type* [30] or significant differences in number, neuroanatomic location or size of brain metastases [31].

Moreover, some authors have suggested a possible interaction between EGFR mutation type and site of metastatization. For instance, Sekine et al. reported that patients harboring exon 19 deletions present a peculiar pattern of brain metastatization that resemble to that of miliary brain metastases, with multiple and small brain tumors with minimal peritumoral edema [32].

In addition to classic “*common mutations*” ~10% of patients with EGFR mutations may ehibit mutations in codons other than 19 and 21. Rare mutations could be detected in a small fraction of patients including exon 18 mutations, predominantly at G719 (~3–4%), exon 20 mutations (~4–5%) as well as uncommon mutations in exon 19 (for example, in-frame insertions) and 21 (i.e. L851Q/R), and finally compound mutations. The clinical significance of these rare mutants is not always fully understood with some variants associated with response to EGFR TKIs (for instance, exon 18 G719X) other associated with resistance (i.e. exon 20 T790M) and other with uncertain clinical significance [33–35]. The mechanism by which these mutations confer resistance to EGFR TKIs is not always fully understood, but a better knowledge of the molecular basis of their activity may help to overcome resistance in patients harboring these mutations [36].

The presence of EGFR mutations defines a specific molecular subgroup of NSCLC (~15% of Caucasian patients with adenocarcinomas) [37, 38] that respond to an EGFR TKI in over 60% of patients (Table 1). The role of these inhibitors in EGFR wild type tumors is instead more debatable, since the benefit in such patient is modest (~8% with Erlotinib in pretreated NSCLC) and no predictive biomarkers have been so far reported in this specific subset of patients [39].

**Table 1:** Phase III trials showing superiority of EGFR TKIs over first-line chemotherapy in EGFR-mutated NSCLC patients

Trial	Selection criteria	Treatment Arms	*N*	RR (%)	PFS (mo)	OS (mo)	References
**IPASS**	East-Asian, light/ non-smoker, adenocarcinoma	Gefitinib vs. Carboplatin/Paclitaxel	132 vs. 129	71.2 vs. 47.3	9.6 vs. 6.3	21.6 vs. 21.9	[17, 63]
**First-SIGNAL**	Korean, non-smoker, adenocarcinoma	Gefitinib vs. Cisplatin/Gemcitabine	26 vs. 16	84.6 vs. 37.5	8.0 vs. 6.3	27.2 vs. 25.6	[64]
**WJTOG 3405**	Japanese, EGFR mutation	Gefitinib vs. Cisplatin/Docetaxel	86 vs. 86	62.1 vs. 32.1	9.2 vs. 6.3	35.5 vs. 38.8	[16, 107]
**NEJ 002**	Japanese, EGFR mutation	Gefitinib vs. Carboplatin/Paclitaxel	114 vs. 114	73.7 vs. 30.7	10.8 vs. 5.4	27.7 vs. 26.6	[44, 108]
**OPTIMAL**	Chinese, EGFR mutation	Erlotinib vs. Carboplatin/Gemcitabine	82 vs. 72	83 vs. 36	13.1 vs. 4.6	22.7 vs. 28.9	[65, 109]
**EURTAC**	European, EGFR mutation	Erlotinib vs. Platinum agent + Gemcitabine or Docetaxel	86 vs. 87	58 vs. 15	9.7 vs. 5.2	19.3 vs. 19.5	[66]
**LUX-Lung 3**	Asian and European, EGFR mutation	Afatinib vs. Cisplatin/Pemetrexed	230 vs. 115	56.1 vs. 22.6	11.1 vs. 6.9	25.8 vs. 24.5	[18, 79]
**LUX-Lung 6**	Asian, EGFR mutation	Afatinib vs. Cisplatin/Gemcitabine	242 vs. 122	66.9 vs. 23.0	11.0 vs. 5.6		[18, 80]

Several phase III trials demonstrated the superiority of EGFR TKIs over chemotherapy in EGFR-mutated NSCLC in the first line setting in terms of overall response rate (ORR) and progression free survival (PFS), but not in overall survival (OS) due to the extensive cross-over between treatment arms, although recently a pooled analysis of patients harboring common mutations enrolled in the LUX-Lung-3 and -6 trials reported a survival advantage also [18]. Since no survival gain was demonstrated in the phase III trials conducted so far, the use of EGFR TKIs in EGFR-mutated patients in first- or second-line should not influence the eventual survival outcome, as demonstrated in the phase II trial of the Spanish Lung Cancer Group [40]. However, retrospective analyses of large phase III trials reported inferior ORR than commonly observed in the front-line setting in EGFR-mutated patients treated with EGFR TKIs after chemotherapy [41–44]. A possible explanation might come from studies that reported an influence of chemotherapy on EGFR mutation status, with lower mutational rate in patients previously treated with chemotherapy [45, 46]. Chemotherapy may selectively kill and inhibit mutant clones within the tumor, whereas *wild type* clones may proliferate, altering the relative proportion of EGFR-mutated/EGFR-*wild type* cells within the tumor mass. A direct observation of increased sensitivity to chemotherapy is the fact that patients with EGFR mutations usually exhibit increased ORR to first-line chemotherapy [47]. These studies underlie another emerging problem, the presence of tumor heterogeneity.

In 2012 in a seminal paper Gerlinger and coll. reported evidence of intratumor heterogeneity and spatial separation of subclones in metastatic renal cancer, establishing the “*branched evolution*” hypothesis [48], suggesting that biomarker sampling in a single tumor region may undetect the complex genomic landscape of a tumor, providing major challenges to clinical implementation of personalized medicine and biomarker development. Two recent papers addressed this issue in NSCLC also. De Bruin et al. reported spatial and temporal tumor heterogeneity of driver mutations within NSCLC, raising the possibility that a single-region sampling biopsy, as commonly done in clinical practice, could miss clinical-relevant driver events present in specific tumor subclones [49]. In contrast, Zhang et al. did not found substantial differences within the same tumor in driver mutations, suggesting that single-biopsy analysis might be sufficient to identify the majority of known driver mutations in lung adenocarcinomas [50]. In addition, a high concordance rate for recurrent somatic alterations has been reported between primary NSCLC and metastases, suggesting that a new biopsy for genomic information to guide treatment decision may not necessary when it is present an archived primary material, since key driver oncogenic events seems to occur early in NSCLC tumorigenesis [51].

Further studies are required to throw a light on this controversial issue and therefore use of a single-site biopsy should continue to be the standard in clinical practice.

### EGFR inhibition in EGFR-addicted NSCLC: A successful 10-year story

The clinical development of EGFR inhibitors in NSCLC started before the discovery of EGFR mutations, therefore initial studies with Gefitinib and Erlotinib were conducted in unselected patients in both pretreated [52–54] and in treatment-naïve patients in combination with standard chemotherapy [55–58]. In attempt to identify patients with improved outcome when treated with EGFR TKIs, several predictive biomarkers, such as EGFR overexpression/amplification [59] and HER2 overexpression/amplification [60], were studied before EGFR mutations emerged as the major predictive factor to these targeted agents. The discovery of EGFR mutations and the dramatic responses observed with EGFR TKIs in retrospective and small phase II studies [20, 61, 62] paved the way to a paradigm shift in the clinical trial design, favoring the development of clinically and then molecularly selected studies in NSCLC. Gefitinib was the first EGFR TKI receiving regulatory approval for EGFR-mutated NSCLC patients after the publication of the results of the IPASS (Iressa Pan-Asia Study) trial, reporting a significant advantage in terms of PFS and ORR for first-line Gefitinib versus Carboplatin/Paclitaxel [63]. Several other trials conducted in Asia confirmed the superiority of Gefitinib over chemotherapy in EGFR-mutated patients [16, 44, 64].

The extension of approval of Erlotinib for patients harboring EGFR mutations, in addition to the previous indication for unselected patients with NSCLC after at least one chemotherapeutic line, was obtained after the publication of two large phase III trials conducted in Asia [65] and Europe [66], providing the definitive demonstration of superiority of an EGFR TKI over standard first-line chemotherapy in molecularly selected patients.

Unfortunately, despite initial responses, virtually all patients, ultimately, progress because of the acquisition of resistance. In addition, a proportion of patients with EGFR mutations exhibits de novo resistance and does not respond to TKIs treatment. Several mechanisms of primary and acquired resistance to EGFR TKIs [67, 68] have been so far reported in *in vitro* NSCLC models and some have also been confirmed in patients. Some of these mechanisms seem to be mutually exclusive, although distinct mechanisms of resistance may be operative in the same tumors [69, 70]. Several strategies have been developed for overcoming acquired resistance to the EGFR TKIs [71, 72] and the use of irreversible, covalent-binding, EGFR TKIs (the so called “*second generation*” inhibitors) has been historically one of the most extensively studied. Despite promising preclinical evidences of activity against EGFR-mutated cell lines harboring the gatekeeper T790M mutation [73–75] the most frequent mechanism of acquired resistance (~50–60% after rebiopsy) [69, 70], neither Afatinib [76] nor Neratinib [77] nor Dacomitinib [78] demonstrated significant activity as single agent in patients harboring the T790M mutations. The failure of this strategy is probably due to the high drug concentrations required for reverting acquired resistance that are not achievable in clinical practice because of inacceptable toxicity, due to the activity of these agents against *wild type* EGFR. Instead, their role is more defined in the front-line treatment of NSCLC patients harboring EGFR activating mutations. Recently, Afatinib received regulatory approval in the first line setting after the publication of the LUX-Lung-3 and -6 trials [79, 80] and another irreversible EGFR inhibitor, Dacomitinib, has reported promising results in a phase II study [81]. Ongoing comparative phase III trials (ARCHER 1050, LUX-Lung-7) will provide definitive evidences whether these agents are superior than first-generation EGFR TKIs (i.e. Gefitinib and Erlotinib) in this clinical setting.

However, the role of second generation EGFR TKIs in acquired resistance is not definitely thrown over, since combining these agents with other targeted agents, such as the dual combination Afatinib plus Cetuxumab [82] and Neratinib plus Temsirolimus [83], has recently reported promising results, albeit toxicity profile of these combinations could limit their clinical implementation. The SWOG 1403 study is ongoing and will evaluate Cetuximab-Afatinib combination in the first-line setting in patients harboring EGFR sensitizing mutations.

In attempt to overcome the limits of second-generation EGFR inhibitors, a novel class of mutant-selective inhibitors has been developed. The first in class third generation inhibitor, named WZ4002, was discovered in 2009 [84], through a functional pharmacological screens against T790M mutant kinases. Unlike other EGFR TKIs that possess a structurally related quinazoline-based core scaffold and were identified as ATP-competitive inhibitors of *wild type* EGFR, WZ4002 is a covalent pyrimidine EGFR inhibitor specifically designed against EGFR T790M that is 30–100 fold more potent against EGFR T790M, and up to 100 fold less potent against *wild type* EGFR than quinazoline based EGFR inhibitors *in vitro* and is effective in murine models of lung cancer driven by EGFR T790M [84]. WZ4002 did not progress into human clinical trials, but three different mutant-selective irreversible inhibitors (Rociletinib, AZD9291 and HM61713) have recently reported *in vivo* impressive activity in patients with acquired resistance to EGFR TKIs. Rociletinib (also known as CO-1686) is structurally related to WZ4002 and is the first drug of its class in clinical development for the treatment of T790M-positive NSCLC. *In vitro* and *in vivo* studies demonstrated that Rociletinib irreversibly and selectively inhibits mutant EGFR, including T790M mutation, with minimal activity against *wild type* EGFR, suggesting that drug tolerability to this agent may be superior to first and second generation EGFR inhibitors, for which major toxicities (diarrhea, skin rash and interstitial lung disease) are attributed to *wild type* EGFR blockage. Interestingly, NSCLC cells resistant to Rociletinib do not exhibit secondary mutations of the EGFR or amplification of the EGFR gene, but increased expression of genes involved in the epithelial-mesenchimal transtition (EMT) and Akt pathway [85]. Recently, Sequist and coll. reported the preliminary results of the ongoing phase I/II study TIGER X in patients with NSCLC harboring EGFR mutations and T790M-positive that previously received an EGFR TKI. The results, albeit preliminary, are quite impressive with a 59% ORR and a median PFS not yet reached, but exceeding 12 months. The safety profile of this agent is also interesting with the most common toxicity represented by hyperglycemia [86]. The expansion phase II cohort of the study is ongoing and three trials (TIGER 1–3) will be launched in different clinical settings. Another third generation EGFR inhibitor is AZD9291. This monoanilino-pyrimidine compound is structurally distinct from other third generation EGFR TKIs and in preclinical studies potently inhibited signaling pathways and cellular growth in both EGFR-mutant and T790M-mutant cell lines *in vitro*, with lower activity against *wild type* EGFR lines, with profound and sustained tumor regression in EGFR mutant tumor xenograft and transgenic models [87]. *In vitro* data suggests the potential to target both HER2 and HER4 kinase activity, a property that may be important as HER2 amplification may mediate acquired resistance to EGFR TKI in 5–12% of cases [70, 88]. AZD9291 also appears to be effective against other rare drug-sensitive EGFR mutants with low activity against EGFR exon 20 insertions [83]. AZD9291 demonstrated promising activity in a phase I study in patients with EGFR-TKI resistant NSCLC with an ORR of 51% and an impressive ORR of 62% in patients T790M-positive, with no dose-limiting toxicities and a maximum tolerated dose not defined [89]. Three ongoing trials are evaluating the activity of AZD9291 in T790M-positive NSCLC (AURA 1–3 trials). Based on these impressive preliminary results the US Food and Drug Administration (FDA) granted the breakthrough designation to both Rociletinib and AZD9291 for patients EGFR T790M-positive NSCLC progressing during an FDA-approved EGFR-TKI.

Preliminary results of a dose-escalating phase I study evaluating a third mutant-selective inhibitor, HM61713, has been recently presented. This inhibitor was well tolerated and reported a promising activity in EGFR-mutated NSCLC patients previously treated with an EGFR-TKI with a more prominent activity in T790M-positive tumors (ORR 29.2%, DCR 75.0%) than T790M-negative (ORR 11.8%, DCR 55.9%) [90]. The expansion part of the study is planned.

### Emerging challenges

#### Brain metastases

The emergence of brain metastases (BM) represent a major issue in clinical practice and is associated with a dismal prognosis. Traditionally, systemic treatments were considered mostly ineffective on BMs, with RR of 23–50% with platinum-based combinations [91]. The low molecular weight, the good toxicity profile and the unprecedented RR and PFS observed make appealing the use of EGFR TKIs in patients harboring EGFR mutations with BMs. Retrospective studies and small phase II trials reported intriguing activity for EGFR TKIs in EGFR-mutated patients with BMs, with RR ranging from 60% to 100% and complete response (CR) rates that ranges 40% [91, 92]. Therefore front-line use of EGFR TKIs in patients with asymptomatic BMs harboring EGFR mutations could be the preferable option in this setting, delaying the use of whole brain radiotherapy [93].

However, the degree of Blood Brain Barrier (BBB) penetration of EGFR TKI may be influenced by factors patients, such as the extent of central nervous (CNS) disease and prior CNS-directed therapies, explaining why the CNS may still act as a partial sanctuary site, with approximately 20% of patients with EGFR-mutant tumors developing BMs while on EGFR TKI therapy [94]. Different strategies have been hypothesized to overcome this inadequate drug exposure, such as high pulsatile dose of EGFR TKI [95] or, in case of oligoprogressive disease, use of TKI beyond progression with the addition of local ablative therapies [96, 97].

The recent results, albeit preliminary, with third generation EGFR inhibitors with responses seen even in the CNS are encouraging [86] and deserve further investigations.

#### Translating the results from advanced setting to adjuvant therapy

The use of EGFR TKIs is the standard of care in advanced/metastatic NSCLC with activating EGFR mutations, however their role in early-stage lung cancer is far less defined. Two large randomized trials evaluated the role of EGFR TKIs in unselected resected stage IB-IIIA NSCLC. The BR.19 study started in 2002 when adjuvant chemotherapy was not considered standard of care in resected NSCLC. Therefore, in 2003 the study was amended to allow adjuvant chemotherapy, so only 17% of patients received adjuvant chemotherapy and 5% adjuvant radiotherapy. The BR.19 randomized patients to receive Gefitinib 250 mg/d versus Placebo for 2 years [98]. After the publication of the dismal results of the SWOG S0023 trial [99], that reported a detrimental effect for Gefitinib maintenance after definitive chemo-radiotherapy the BR.19 study was prematurely closed. The results of the study demonstrated not only no an advantage in both DFS and OS in the overall population and in the small subgroup of EGFR-mutated patients (4%), but also a possible detrimental effect.

The detrimental effect observed even in EGFR-mutated patients may be attributable to the low mutation rate observed in the study, that could not have a sufficient powerful effect on the study results [98]. The results of the RADIANT trial were recently reported. In this study 973 patients with resected stage IB-IIIA NSCLC were randomized to either Erlotinib 150 mg/d or placebo for 2 years with or without adjuvant chemotherapy. The study failed to demonstrate a DFS advantage in the overall population (50.5 months vs. 48.2 months), but a trend toward a longer DFS was observed in the subgroup of patients with EGFR mutation (16.5%), albeit not statistically significant (46.4 months vs. 28.5 months) (HR 0.61, 95% CI 0.38–0.98, *p* = 0.039) [100, 101]. Moreover, a retrospective study of the Memorial Sloan-Kettering Cancer Center reported a significant reduction of risk of recurrence or death (HR 0.43, *p* = 0.001) and a trend toward improved OS (HR 0.50, *p* = 0.076) for patients with resected NSCLC treated with Erlotinib or Gefitinib with or without adjuvant chemotherapy [102].

Ongoing studies in resected EGFR-mutated NSCLC (NCT01405079; NCT01410214; WJOG6410L; NCI-2014–01508) will definitely provide evidence of efficacy of this strategy, as observed in other solid malignancies, such as Imatinib in GIST and Trastuzumab in HER2-positive Breast Cancer.

#### Uncommon and resistance EGFR mutations

Exon 19 in-frame deletions and point-mutation L858R of the exon 21 account for more than 90% of all EGFR mutations and are associated with sensitivity to EGFR TKIs.

However, efficacy data for the remaining ~10% of EGFR mutations are lacking and mostly from retrospective analyses [34, 103]. Data from the LUX-Lung project were recently presented, showing the activity of Afatinib in the largest prospective dataset in patients with rare mutations. The picture emerging from this analysis is complex with high heterogeneity among this peculiar molecular subgroup. Patients with *de novo* T790M mutations or exon 20 insertions presented in general inferior ORR and shorter PFS and OS compared with patients harboring other uncommon mutations, such as exon 18 G719X and exon 21 L861Q, that exhibited a range of sensitivity similar to that of common mutations. However, in some cases with exon 20 insertions or T790M mutations durable control of the tumor was observed [104]. Data from the French ERMETIC-IFCT network reported similar findings, with poorer outcome for patients harboring exon 20 insertions, with the exception of more proximal mutations, compared with complex mutations and exon 18 mutations [105].

A more extensive comprehension of the mechanisms underlying EGFR TKI-sensitivity of these mutations should help to provide more effective strategies for patients harboring these rare mutants. Recently, Yasuda et al. described the chemical structure of the exon 20 mutations D770_N771insNPG, showing that the three amino-acid insertion forms a “*wedge*” that lock the C helix of the EGFR in its inward, active position. Therefore, the clinical resistance of this mutant is not due to steric hindrance with inhibitor binding, but simply not sensitizing to EGFR TKIs inhibition [36]. These findings may help through the screening of compound libraries identification of mutant-selective inhibitors as with T790M-mutants. Moreover, it was demonstrated that exon 20 mutations (EGFR D770_P772del_insKG and D770 > GY), which are intrinsically associated with resistance to EGFR TKIs, activate EGFR by increasing the attractive electrostatic dimerization energies. This might provide the rationale for the use of anti-EGFR monoclonal antibodies in patients with such mutations, since cetuximab, by preventing receptor dimerization, may be associated with therapeutic activity [106].

## CONCLUSIONS

Since the discovery of EGFR mutations in 2004, the therapeutic landscape of advanced NSCLC has dramatically changed with unprecedented results in this usually disappointing disease. However, the clinical success of this strategy is limited by several challenges with which we are called to deal, such as emergence of acquired resistance or treatment of rare and compound mutations. To date three different EGFR TKIs have been approved and no prospective data are available to guide optimal treatment selection in clinical practice. Ongoing comparative randomized studies between reversible and irreversible EGFR TKIs and the recent development of novel mutant-selective inhibitors in patients with acquired resistance will definitely provide definitive conclusions about the optimal therapeutic strategy in this molecularly selected subgroup of patients. Probably, there is no *magic bullet* that can cure cancer by blocking a single signaling pathway, however combining these inhibitors each other or with other no overlapping drugs and a better comprehension of inherited mechanisms of resistance to these targeted agents might provide more durable therapeutic successes with the hope, in a not so far future, to cure lung cancer.
